# Dataset with press forming results of unidirectional thermoplastic composite laminates including in-plane deformation data for validation of forming simulations

**DOI:** 10.1016/j.dib.2024.110099

**Published:** 2024-02-02

**Authors:** Dennis Brands, Václav Vomáčko, Wouter Grouve, Sebastiaan Wijskamp, Remko Akkerman

**Affiliations:** a Production Technology, Faculty of Engineering Technology, University of Twente, Drienerlolaan 5, 7522 NB Enschede, the Netherlands; b ThermoPlastic Composites Research Center (TPRC), Palatijn 15, 7521 PN Enschede, the Netherlands; c Institute of New Technologies and Applied Informatics, Faculty of Mechatronics, Informatics and Interdisciplinary Studies, Technical University of Liberec, Studentská 2, 461 17 Liberec, Czech Republic

**Keywords:** Carbon fiber, Manufacturing, Experiment, Photogrammetry, Wrinkling

## Abstract

Truncated hemisphere parts were press formed with two commercially available unidirectional thermoplastic composite materials, namely Toray TC1225 and Solvay APC. The width and layup of the laminates were varied to influence the wrinkling severity, to trigger various deformation mechanisms and to influence the amount of in-plane deformation. A total of eight layup/width combinations were selected and formed in triplicate for both materials, resulting in the analysis of 48 parts in total. The wrinkling defects are clearly observed due to an intentional gap between the forming tools at the end of forming. Further, a dot pattern with a resolution of 3 mm was applied to the laminates prior to forming using a photoresist mask, sandblasting and heat resistant spray paint. The locations of the dots before and after forming were measured using photogrammetry and are provided in the dataset as a triangular mesh including a precision metric. Matlab functions, bundled with this dataset, allow for the reproduction of the deformation calculations and averages. Lastly, a Matlab App (GUI) is provided for easy visualization of the data. This dataset can serve as a reference for validation of composite forming simulations.

Specifications Table

**Table:** 

Subject	Materials science: Ceramics and Composites, Polymers and Plastics
Specific subject area	Press forming of unidirectional fibre reinforced thermoplastic composites.
Data format	Raw, Analysed, Averaged
Type of data	Images (.png/.jpg file), Triangular mesh (.msh file, ASCII text), Table (.csv file)
Data collection	The composite laminates were consolidated and press formed using the 200t press from Pinette Emidecau Industries at the ThermoPlastic composites Research Center in Enschede. A standard water-cooled diamond saw was used for rough cutting the laminates and a Datron Neo milling machine for final dimensioning. Rayzist SR3000 photoresist mask was used in combination with conventional grid blasting and MoTip heat resistant silver paint to apply the dot pattern. A Nikon D5600 digital camera with an AF-S 50mm lens was used to make photos, followed by the photogrammetry software PhotoModeler 2023 for digitization of the dot patterns. Subsequent analyses and calculations were performed with Matlab 2021b.
Data source location	ThermoPlastic composites Research Center (TPRC), Enschede, the Netherlands
Data accessibility	Repository name: 4TU.ResearchData Data identification number: 10.4121/80766aa2-d92c-420b-9ec4-bad1b9e4d927 Direct URL to data: https://data.4tu.nl/datasets/80766aa2-d92c-420b-9ec4-bad1b9e4d927

## 1 Value of the Data

• The high-resolution in-plane deformation measurements and clear wrinkling observations allow a comprehensive validation of the finite element models and constitutive relations for composite forming simulations.
• Eight varieties of forming configurations were realized by changing the layup, blank geometry and process parameters. All configurations have been formed using both materials considered.
• Every unique forming configuration was formed in a set of three replicates to provide insight into the variability of the material and process. The average deformation was also calculated for more reliable comparison against simulation models.
• The dataset provides composites engineers and designers insight into the formability of two commercially available and frequently used unidirectional thermoplastic composite material systems.

## 2 Data Description

The following naming convention was used for the press-formed parts: “C-LMP 40 [0, 45, 90, -45]s L1A”, where: “C-LMP” is material, “40” is width of specimen in [mm], “[0, 45, 90, -45]s” is layup, “L1” is laminate number and “A” is position in laminate (A: left, B: middle, C: right, see Fig. 3).

The dataset has following structure:

**01_Photogrammetry_Flat**

Has subfolders for each of the 48 parts formed, each with 16 pictures used for the photogrammetry analysis of the undeformed state.

**02_Photogrammetry_Formed**

Same as “01_Photogrammetry_Flat”, however, containing pictures of the deformed part state.

**03_Individual**

Contains meshes and deformation data for all 48 unique parts in their respective subfolders. Each subfolder contains the same set of files. Flat and formed meshes in “.msh” (ASCII text, native to the GiD preprocessing software) file format are the result of the photogrammetry analysis. Both have corresponding files with precision data, provided as standard deviation on the nodal positions, in “.csv” format. The files named “result_*.csv” are the outcome of the deformation calculations. Nodal averaged and element values are provided for: fiber direction (“_fiber”), Green-Lagrange strain (“_glstrain”) and shear angle (“_shearangle”). Lastly, files named “precision_*.csv” supply the standard deviation values on the deformation calculations, propagated from the standard deviation on the nodal coordinates using Monte-Carlo simulation.

**04_Average**

Parts were formed in sets of three replicates, each subfolder contains the averaged deformation data for such a set. Folder structure follows the same naming convention as previous. Mean (“_mean”) and standard deviation (“_std”) values of the deformation are provided as nodal average and element value for fiber direction, Green-Lagrange strain and shear angle. Meshes for the undeformed (“flat.msh”) and deformed (“formed.msh”) laminates are provided for each of the three individual parts, only including the nodes that were present in all three of the original deformed meshes.

**05_Photos_Parts**

Photos after press forming from top and bottom view of all 48 specimens.

**Fig. 1:**
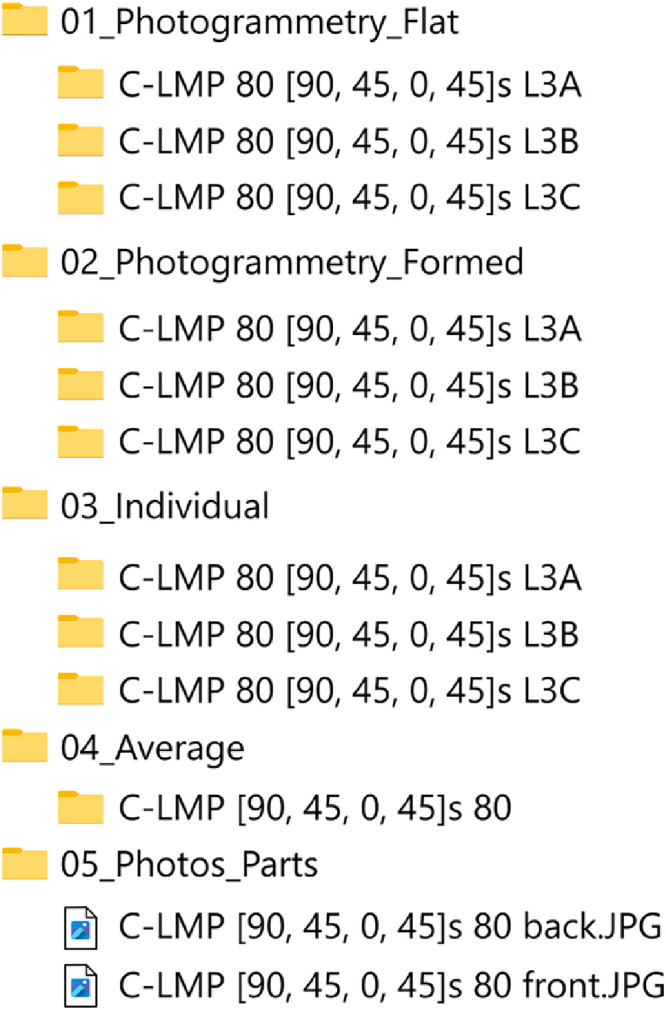
Example of subfolders and files that belong to the results from the same test configuration. The example shows TC1225 material, 80 mm width and a [90/-45/0/45]_s_ layup.

The previously mentioned folders (01-05) contain the main data from the experiments. Fig. 1 illustrates how the subfolders and files correspond between these folders. The remaining folders (06-09) contain additional tools or metadata.

**06_Code**

Matlab code files (ASCII text) used to calculate deformations and average data.

High-level functions:

- “*loadResultFolder.m*” loads the data from a subfolder of “03_Individual” or “04_Average” into a structure for subsequent analysis.
- “*computeDeformation.m*” is used to calculate the deformation for a subfolder of “03_Individual”. It utilizes the files “flat.msh” and “formed.msh”.
- “*computeAverage.m*” can calculate the average deformation based on a set of three result structures as obtained using “*loadResultFolder.m*” or “*computeDeformation.m*”. In cases with severe wrinkling, the meshes are reduced to the set of elements where all nodes are present in each of the three input results.

Low-level functions:

- “*readGiDMesh.m*” reads a file in the “.msh” format.
- “*loadDeformationState.m*” loads the contents of a “.msh” file into a structure, including precision data, if available.
- “*computeElementDeformation.m*” is used to calculate deformations on elements between two meshes, based on a definition of two initial fiber directions.
- “*extrapolateToNodes.m*” is used to perform nodal averaging of the element data obtained using “*computeElementDeformation.m*”.
- “*getFiberDefinitionByName.m*” provides definitions for the initial fiber directions based on the name of the folder/part.
- “*getConfigFolders.m*” is a function to map between the subfolders in “03_Individual” and the subfolders in “04_Average”.

**07_ResultViewer**

**Fig. 2:**
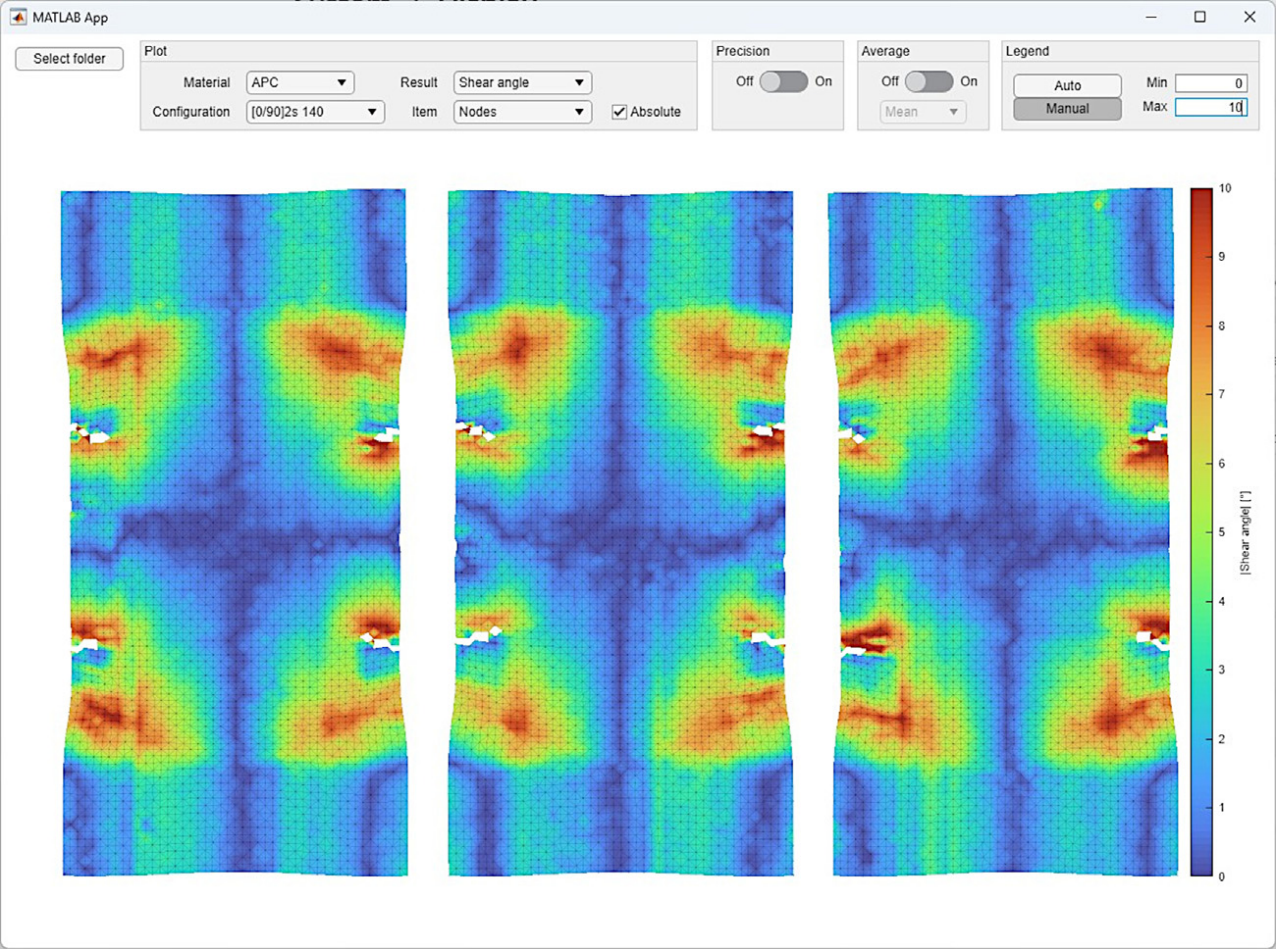
Example screenshot of the ValidationResultViewer application.

Application ValidationResultViewer is provided to easily plot the data provided in the folders “03_Individual” and “04_Average” using a convenient Matlab user interface. A screenshot of the app is shown in Fig. 2.

**08_Photos_Process**

Photos and videos of the press forming process and specimen preparation.

**09_PI_Tape_characterization**

Complementary tensile test data on the polyimide tape that was used in the forming process. A subfolder “Raw data” contains “.csv” files with the raw measurement data of each specimen and a Matlab script that produces the averaged and analyzed results. The analyzed data is supplied as an image, see Fig. 12, and as tabular data in a “.csv” file. Images of the setup and specimens provide a more visual understanding of the experiments.

## 3 Experimental Design, Materials and Methods

### 3.1 Materials

Two types of unidirectional carbon fiber reinforced thermoplastic composite tapes were examined: Toray TC1225 and Solvay APC. Important datasheet characteristics on these materials are collected in Table 1. The Toray TC1225 material is referred to as “C-LMP” in the dataset. Solvay APC is referred to as “C-PEKK”.

**Table 1:** Material characteristics.

	Toray TC1225	Solvay APC
Fiber	T700	AS4D
Fiber areal weight [g/m2]	145	145
Fiber volume fraction [%]	59	59
Matrix	LM-PAEK	PEKK-FC
Resin content by weight [%]	34	34
Glass transition temperature [°C]	147	159
Melting temperature [°C]	305	337
Consolidated ply thickness [mm]	0.14	0.14

**Fig. 3:**
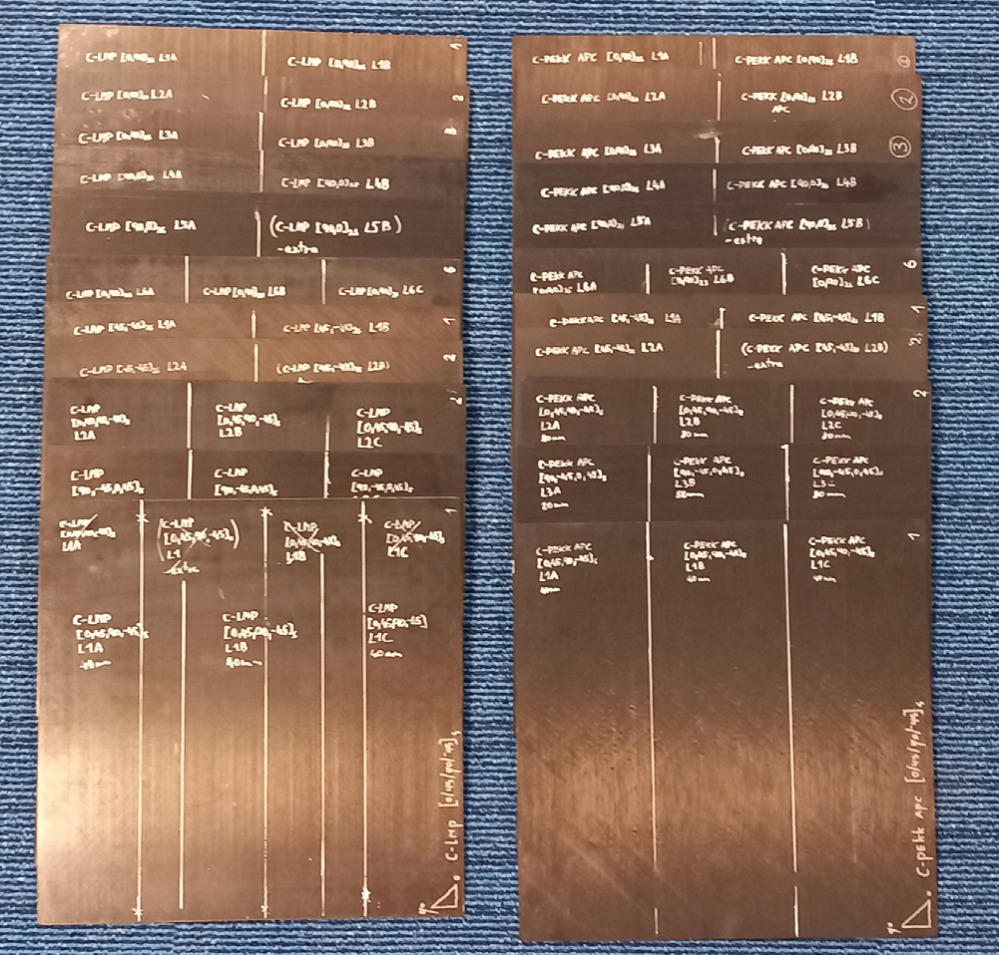
All laminates used for forming, with rough making for individual blanks.

Single plies were cut from the roll of material. Plies were then loosely stacked inside a 12-inch picture frame mold according to the desired layup. Three laminates were stacked in the same mold and were separated by 1 mm thick steel caul sheets; release agent was applied to all tooling surfaces. Solvay APC was consolidated at 375°C, 10 bar for 15 minutes and Toray TC1225 at 365°C, 10 bar for 30 minutes. The heating rate was 10°C/min, while cooling rate was 5°C/min for both materials. Blanks were cut to rough dimensions using a water-cooled diamond-coated blade. Fig. 3 shows the laminate before cutting, with markings for rough dimensions and blank labels.

### 3.2 Test Matrix

Eight configurations with differences in layup, blank width and process settings were selected and are presented in Table 2. Each configuration was performed in 3 repetitions for both materials, giving a total of 48 parts in the test matrix.

**Table 2:** Overview of press configurations. The 0°-direction is aligned with the length direction of the blank. The configuration marked with * used a slower forming speed. Length is 295 mm in all cases.

Layup	Width [mm]
[0/45/90/-45]_s_	40
[0/45/90/-45]_s_	80
[90/-45/0/45]_s_	80
[0/90]_2s_	80
[0/90]_2s_	140
[0/90]_2s_*	140
[90/0]_2s_	140
[45/-45]_2s_	140

### 3.3 Pattern Application

**Fig. 4:**
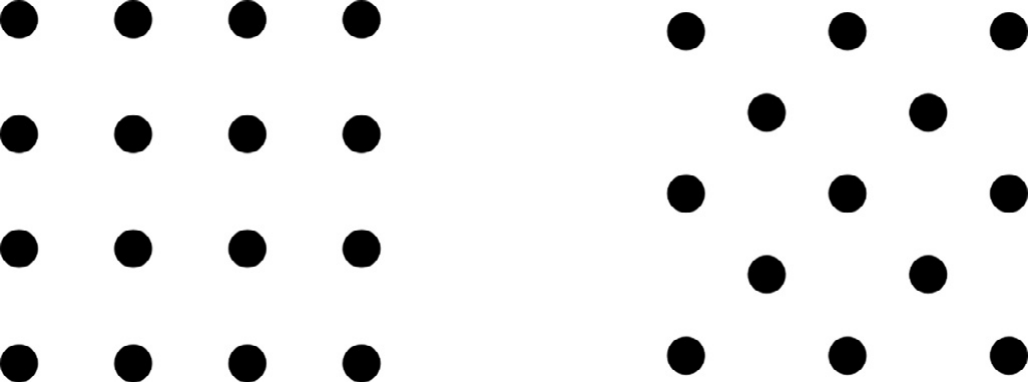
Dot patterns with dot size 1 mm and 3 mm spacing, for 0/90 orientation (left) and 45/-45 orientation. Note that images are not to scale.

To track deformation during the stamp forming process, a dot pattern was used. This pattern was applied with the use of a mask followed by a combination of sandblasting and spray paint. The dot size was 1 mm with 3 mm spacing on a regular grid that is aligned with the fiber direction on the outer ply, hence the dot pattern is under a 45-degree angle for the [45/-45]_2s_ layup, see Fig. 4.

**Fig. 5:**
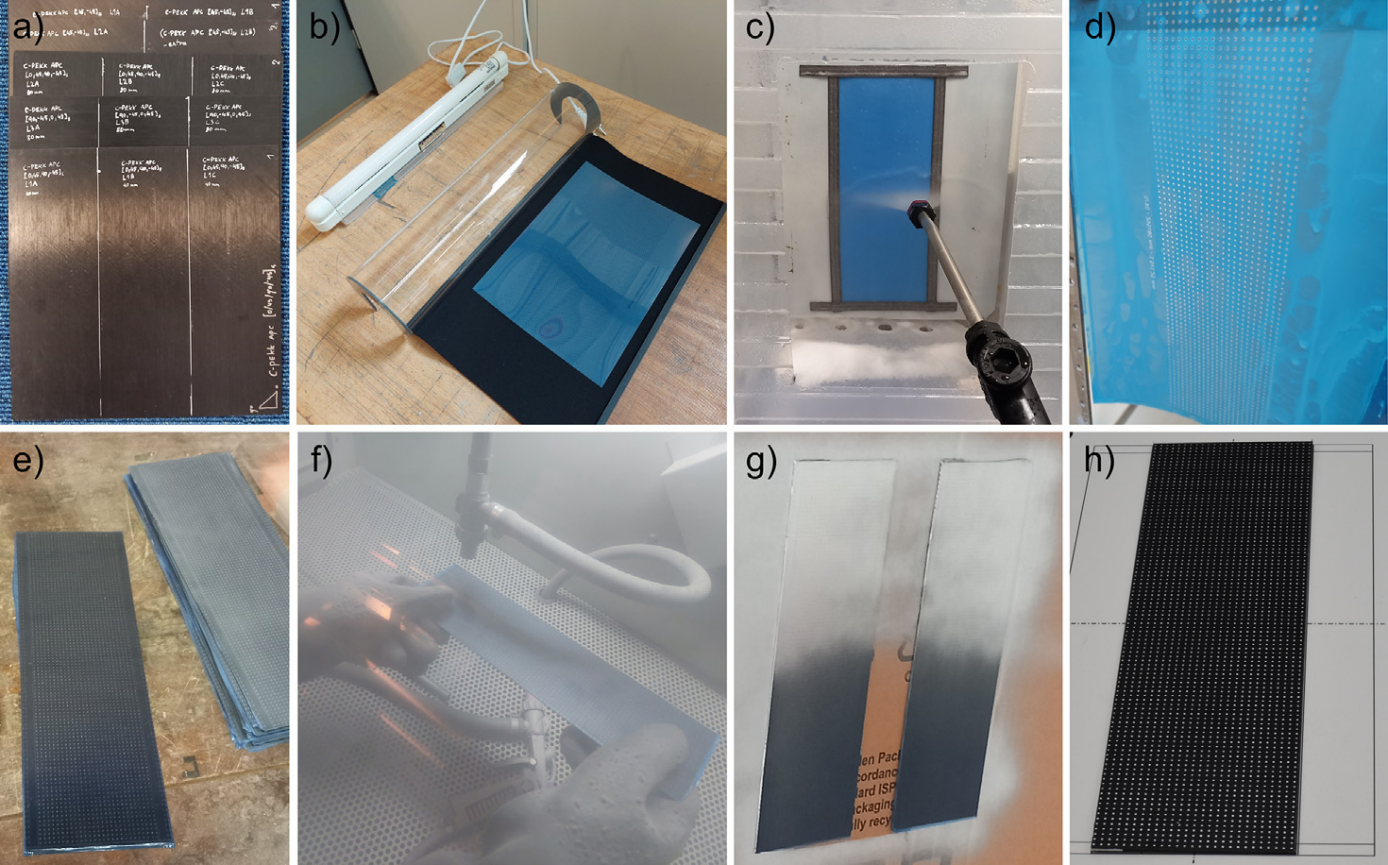
Steps in blank preparation. (a) Consolidated laminates. (b) Photoresist exposure. (c) Photoresist washing. (d) Photoresist drying. (e) Mask applied onto laminate. (f) Sandblasting. (g) Painting. (h) Photogrammetry.

The sandblasting mask was created using Rayzist SR3000 photoresist film and the tools in the StarterSet BULBY SR3000 [1]. A visual overview of this process is shown in Fig. 5. First the dot pattern was printed on a transparent Inkjet media exposure mask, which is used to transfer the pattern onto the photoresist film through UV light exposure for 20 seconds. The areas exposed to UV light harden and the unexposed areas (dot pattern) were washed out using a pressure washer. Afterward, the photoresist masking film was dried before applying it to the laminates, ensuring good alignment of the pattern with the fiber direction of the top ply.

Before painting, the masked laminates undergo a sandblasting step to expose the laminate surface underneath the pattern. The sandblast step is essential to remove a thin polymer film that covers the mask. The laminates were subsequently cleaned with isopropyl alcohol before a heat-resistant silver spray paint (MoTip) was applied. After sufficient drying time at room temperature, the mask is removed for a final cure of the paint at 160°C for 30 minutes.

The painted laminates were then milled to final size of 295 x (140, 80, 40) mm on a Datron Neo milling machine to ensure accurate dimensioning and proper centering of the pattern.

### 3.4 Photogrammetry

Prior to press forming, photogrammetry was used to digitize the dot pattern of the flat, undeformed, blank.

**Fig. 6:**
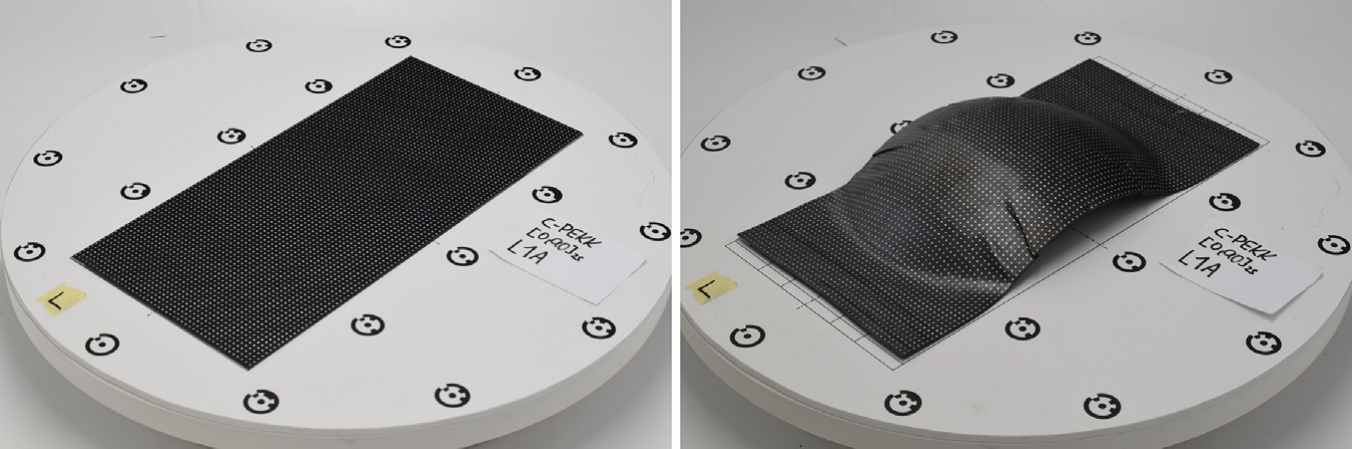
Example of photos for photogrammetry, before and after forming.

First, the part was photographed in a specially designed photography setup featuring adequate lighting, a remote-controlled turntable, a surface with reference marks, and polarizing light filters to eliminate undesired specular reflections. A Nikon D5600 digital camera equipped with an AF-S 50mm lens was employed, utilizing a small aperture (f/16) and a low ISO setting (ISO 100) to ensure a wide depth of field and high image quality. Each part was photographed from 16 equidistant angles around it at a fixed elevation. Example photos can be seen in Fig. 6.

Subsequently, these photographs were analysed using the photogrammetry software PhotoModeler [2]. Automatic point detection and referencing was used to process individual photographs. Then, the software can optimize for the camera orientation and parameters to determine the locations of each dot in 3D space, including a confidence region. A reference length is used to scale the point cloud to real world length units. The reference lengths are defined as 6 pairs of coded targets on the circumference of the base sheet of rotating table with a diameter of 370 mm.

After press forming, the photogrammetry analysis is performed in the exact same manner to digitalize the dot pattern on the formed parts.

### 3.5 Press Forming

**Fig. 7:**
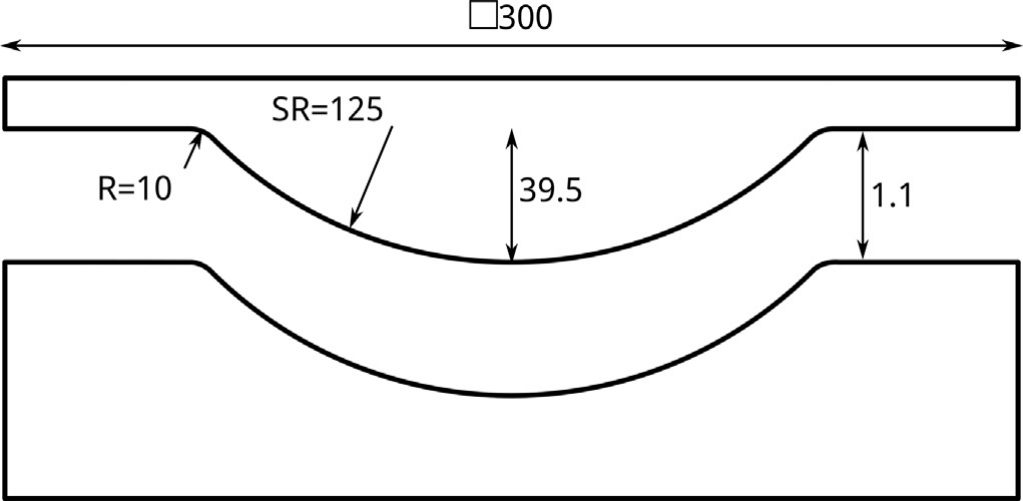
Schematic tooling cross-section. Dimensions in millimetres.

Prior to press forming, the laminates were dried at 120°C overnight. Press forming experiments were carried out using a 200-ton press from Pinette Emidecau Industries installed at the TPRC. For this purpose, a steel hemispherical tool set, as schematically shown in Fig. 7, was utilized. This tool set comprises a convex (male) tool with a radius of 125 mm and a height of 39.5 mm, centred on the 300 mm square tool body. The concave (female) tool maintains a constant offset of 1.1 mm, specifically designed for the nominal thickness of the 8-ply blanks. The tools were was treated with Marbocoat 227CEE release agent prior to heating them to a constant 220°C tool temperature.

**Fig. 8:**
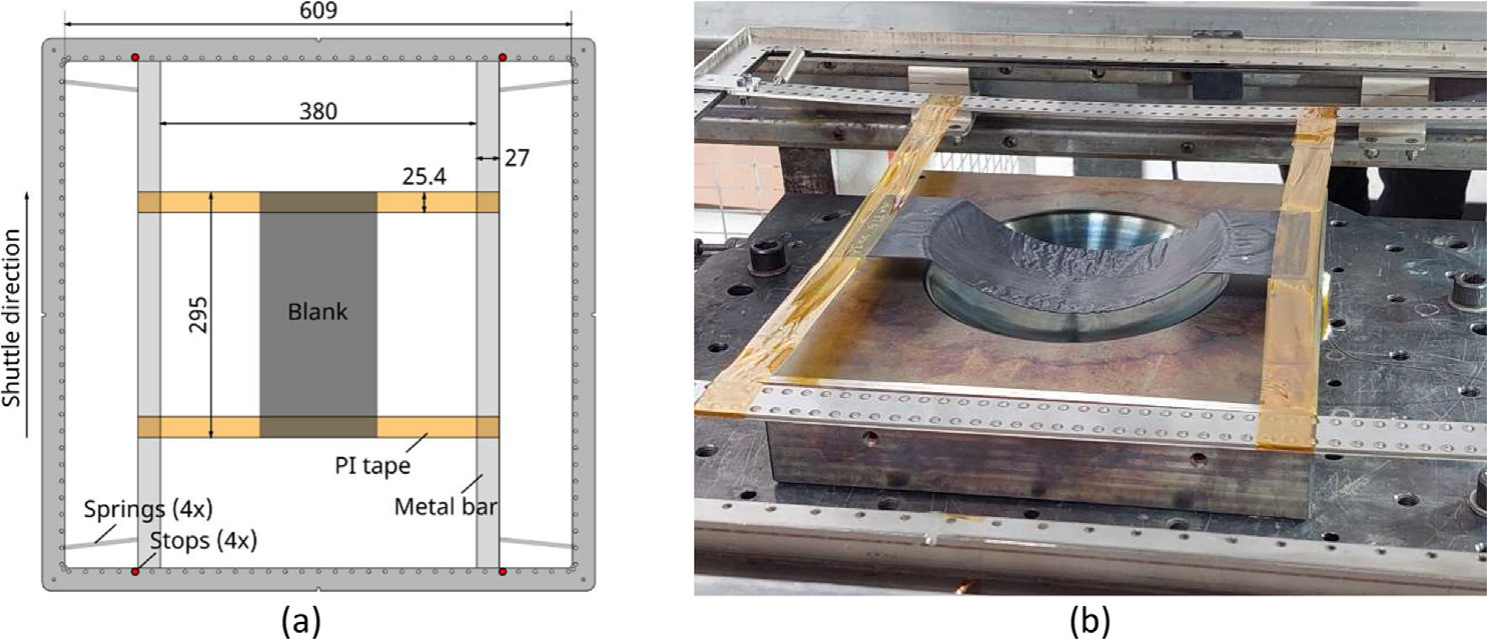
Laminate handling setup. (a) Schematic overview. Dimensions in millimetres. (b) Suspension in shuttle frame after press forming.

The blanks were placed in a shuttle frame to enable automatic transport between the infra-red oven and the press. A suspension system was set up inside the shuttle frame to handle the laminate during transportation between heating and forming. This system consists of two layers of 1-inch-wide polyimide (PI) tape stretched between sliding metal bars, which are tensioned using springs, as shown in Fig. 8. The bars are spaced 380 mm apart and the springs have a stiffness of 0.23 N/mm with a preload of approximately 2 N. The ends of the blanks were encapsulated between the PI-tape, which provided sufficient support during heating and transport. The use of PI tape for suspension ensured that the entire blank could be uniformly heated and fully molten, a requirement since the blanks were smaller than the tool dimensions.

**Fig. 9:**
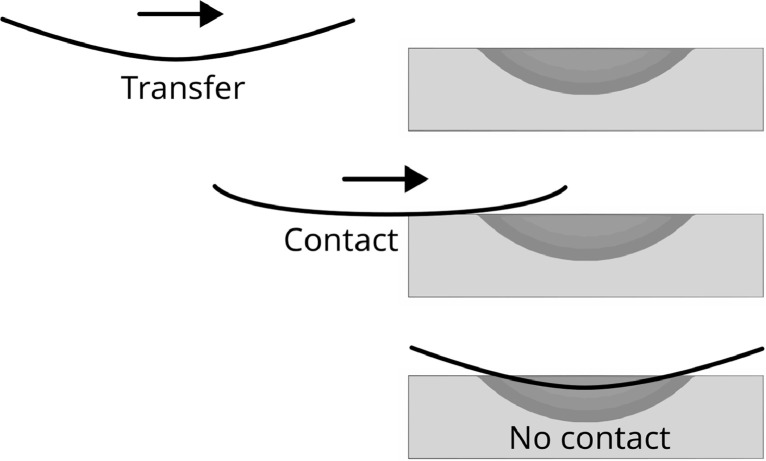
Schematic cross-sections of the blank transfer from infrared oven to press. Black line represents the approximate shape of the laminate.

The blanks were heated to approximately 365°C and 375°C for Toray TC1225 and Solvay APC, respectively. A fixed heating time of 1 minute and 30 seconds ensured consistent heating. Automatic transfer from IR oven to press over a length of 80 cm took 1.4 seconds, with an additional 0.3 seconds before the upper tool started to move. The frame was placed approximately 30 mm above the fixed concave bottom tool, with the blank centred over the tool. Leaving the oven, the blank has deformed due to gravity, leading to contact with the tool as the blank gets dragged over and into the cavity of the bottom tool. This is schematically shown in Fig. 9. However, at the end of transfer, just before to the forming process, there is no longer contact with the bottom tool.

Next, the press closes to form the part. The initial gap between the tool surfaces was 110 mm, with the upper convex tool moving downward. For the majority of the parts the tool closes quickly until it switches to 20 mm/s at a tool gap of 10 mm. The first quick 100 mm stroke takes approximately 1 second to complete. For the parts with the slower configuration the switch is at 43 mm tool gap, just before the tool touches the laminate, and continues to close with 8 mm/s. In both cases the press was not fully closed, hence no consolidation pressure was applied. A final tool gap of 3.1 mm remains, which is 2 mm above the nominal laminate thickness, to observe the out-of-plane deformation of the blank more clearly. All specimens were formed on two days (one day for each material) with exactly the same configuration of the press set-up.

### 3.6 Deformation Calculations

Calculations were performed the same as in a previous publication by Brands et al. [3], but they are briefly summarised here for completeness.

The digitized dot patterns in the undeformed configurations are meshed with a regular triangular mesh. The node numbering between configurations is then made consistent so the same mesh can be applied in the deformed configuration also. For some parts the wrinkling is so severe that not all points can be recognized, hence they are omitted as well as the elements they connect to.

**Fig. 10:**
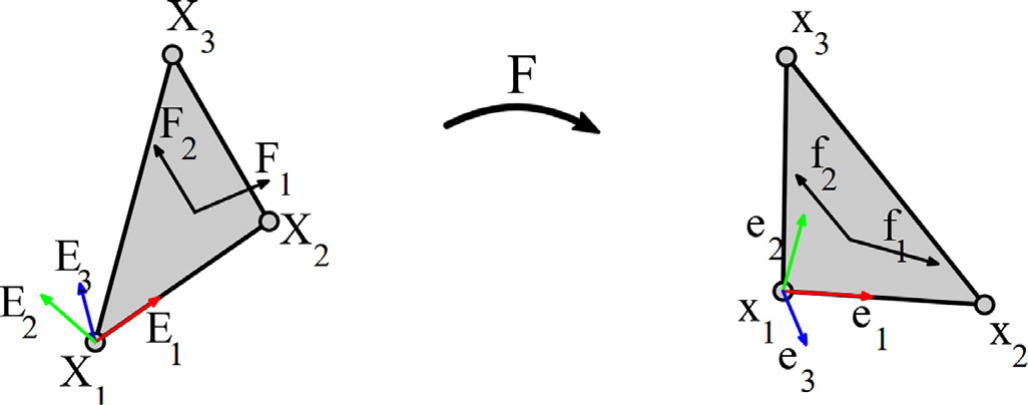
Vector definitions in initial (left) and deformed (right) configurations. Nodal locations in the undeformed (X_1_,X_2_,X_3_) and deformed (x_1_,x_2_,x_3_) configurations connected by a single element. The local coordinate frames indicated with (E_1_,E_2_,E_3_) and (e_1_,e_2_,e_3_) respectively. Finally the fiber directions given by (F_1_,F_2_) (perpendicular) and (f_1_,f_2_) lie in the plane of the element. The deformation gradient tensor **F** maps from the initial to the deformed configuration. Reprinted from [4].

The deformation is assumed to be homogeneous over one element so that linear interpolation can be used. The deformation gradient tensor F can be readily calculated from the nodal locations in the local coordinate system [4], see also Fig. 10. The fiber directions are defined on the undeformed configuration, simply based on the direction of the outer ply. The deformation gradient is then used to calculate the fiber direction in the deformed configuration:

(1) $$f_{1}=\;F\cdot F_{1}\;\text{and}\;f_{2}=\;F\cdot F_{2}$$

The shear angle, i.e. the change in inner fiber angle, is calculated from the fiber directions using:

(2) $$\gamma =\;cos^{-1}\left(f_{1}\cdot f_{2}\right)-\;cos^{-1}\left(F_{1}\cdot F_{2}\right)\;$$

Additionally, the full in-plane Green-Lagrange strain tensor is obtained using:

(3) $$E=\;\frac{1}{2}\left(F^{T}\cdot F-\;I\right)$$

where I is the second-order identity tensor. Coordinate transformation is applied to align the strain tensor with the first fiber direction. Lastly, component-wise nodal averaging is applied to calculate all deformation results at node locations.

### 3.7 Polyimide Tape Characterization

The tensile stiffness of the polyimide (PI) tape used in the forming process was measured at elevated temperatures. This data is complementary to the forming results since it could be used to set-up representative boundary conditions in simulations for the forming processes considered in this dataset.

**Fig. 11:**
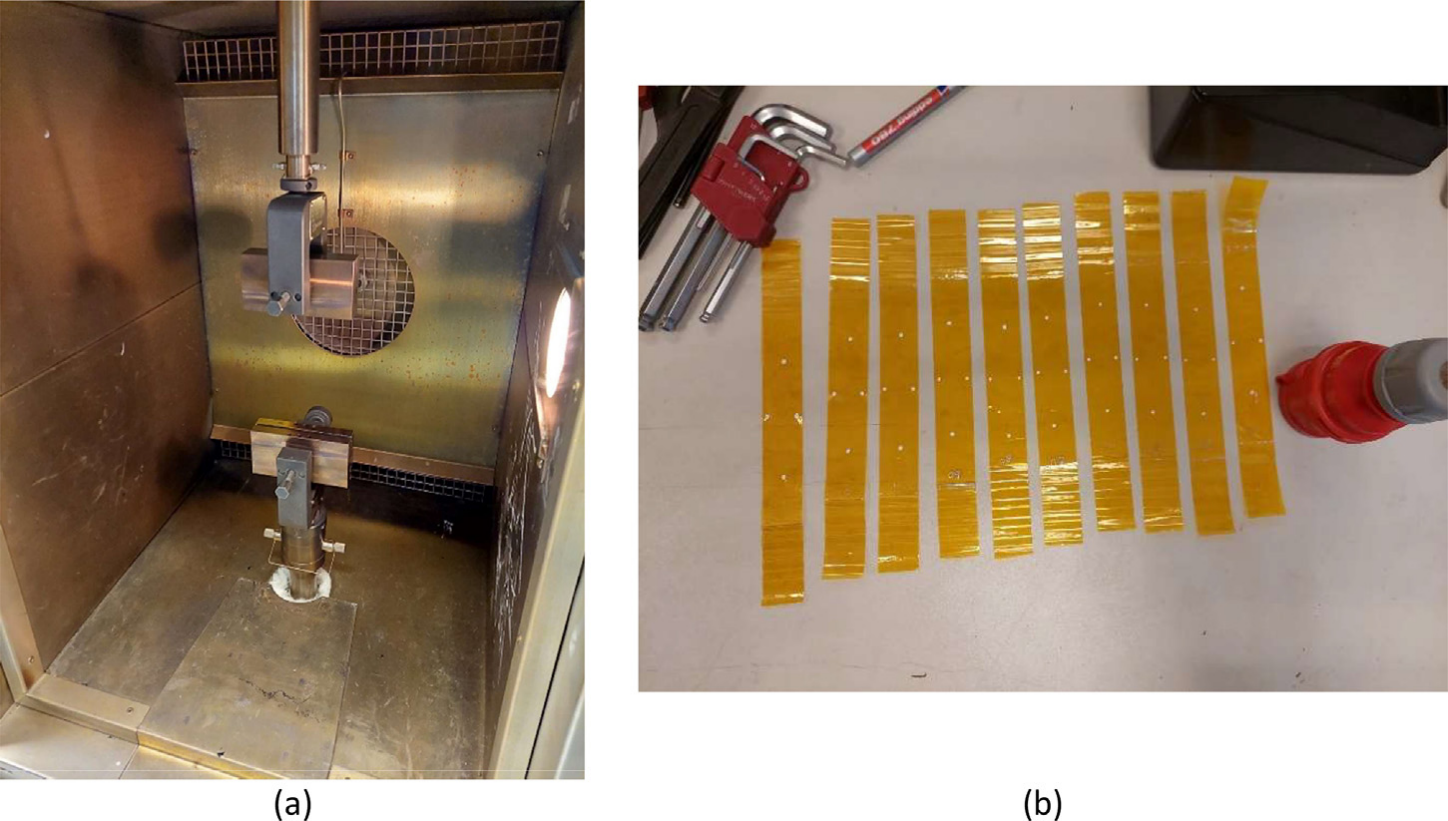
(a) Climate chamber and clamps used in the tensile tests for the PI tape. (b) PI tape tensile specimens after testing.

Two pieces of Airtech Air Kap 136 tape were placed on top of each other, with the adhesive sides bonding them together, see Fig. 11b. The specimens are 200 mm in length, 25.4 mm (1 inch) wide and 0.114 mm thick. Dots are applied to enable the use of a video extensometer to measure longitudinal and transverse strain. The specimen is placed between serrated clamps, see Fig. 11a, with a gauge section of 125 mm, inside a preheated climate chamber. After placement, the oven was brought back up to temperature and an additional 1 minute dwell time was used. Before the start of the test, a 5 N preload was applied to straighten the foil, whereafter the strain values were zeroed. A constant clamp displacement of 2 mm/min was applied up to a longitudinal strain of 2.5%. Tensile tests were performed at room temperature, 100°C, 200°C and 300°C.

**Fig. 12:**
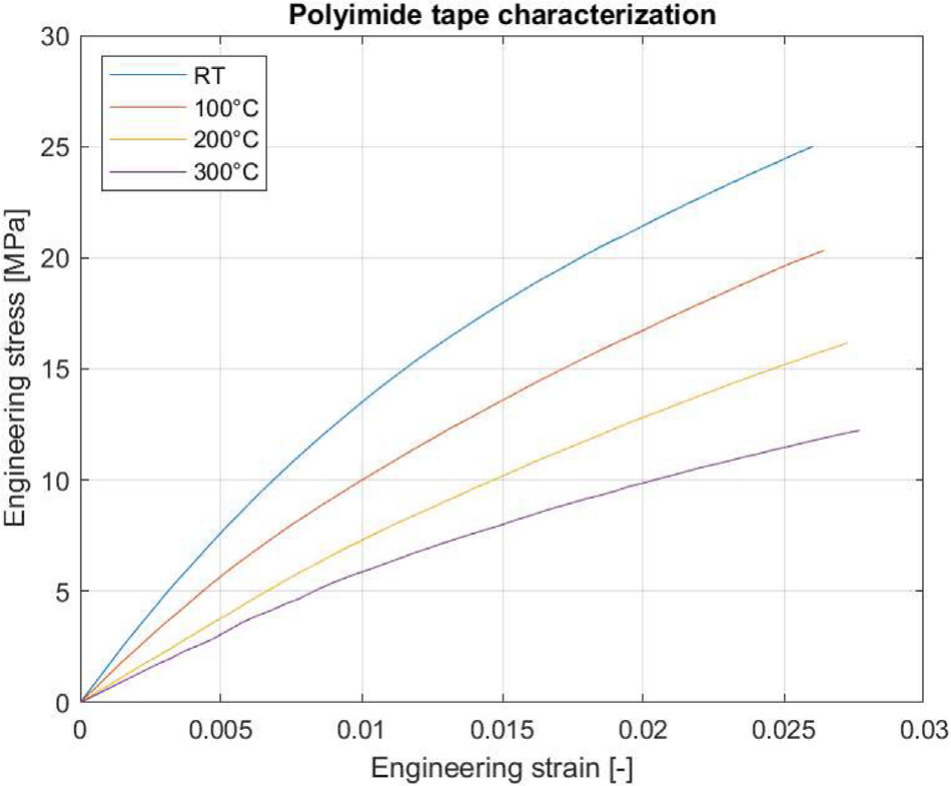
Engineering stress-strain curve for the polyimide tape tested at four temperatures. Raw data was averaged and offset to obtain the curves shown.

The raw data was averaged based on individual temperatures, followed by an offset of the strain to compensate for the applied pre-load. The obtained engineering stress-strain curves are shown in Fig. 12.

## 4 Collection Referral

The dataset presented in this article is part of a collection [5] related to the characterization, modelling and validation of composite forming simulations as part of the “PrEss foRming without deFECTs” (PERFECT) project. The interested reader can find additional information regarding the characterization, modelling and forming behavior of the materials used in this study in the various datasets of this collection.

## Limitations

Great care was put into proper heating of the blank and tools prior to forming. However, no thermocouple data or temperature distributions were obtained during the experiments. Inhomogeneous heating may have had an effect on the forming behaviour. Preliminary experiments with thermocouples did show consistent heating to within +/- 5°C from the setpoint in the centre of the blank at the start of transfer. However, the side of the blank facing the exit of the oven was approximately 5°C lower. The areas underneath the tape were also about 5°C cooler than adjacent areas without tape. The temperature loss due to transfer is estimated to be 5°C also. Through-thickness temperature gradients are assumed to be negligible prior to tool contact. Laminate cooling due to the brief premature tool contact during transfer, as mentioned before, is unknown.

## Ethics Statement

The authors declare that this work adheres to ethical publishing standards and does not include human studies, animal experiments or data collected from social media platforms.

## CRediT authorship contribution statement

**Dennis Brands:** Conceptualization, Methodology, Investigation, Formal analysis, Validation, Software, Writing – original draft. **Václav Vomáčko:** Methodology, Investigation, Formal analysis, Validation, Writing – original draft. **Wouter Grouve:** Supervision, Writing – review & editing, Project administration, Funding acquisition. **Sebastiaan Wijskamp:** Supervision, Writing – review & editing. **Remko Akkerman:** Supervision, Writing – review & editing, Funding acquisition.

## Data Availability

Press forming results including deformation measurements for unidirectional thermoplastic composite laminates (Original data) (4TU.ResearchData).
